# Case Report: Superficial Circumflex Iliac Artery Perforator Flap for Tongue Reconstruction Following the Hemiglossectomy

**DOI:** 10.3389/fsurg.2021.692331

**Published:** 2021-11-04

**Authors:** Yihong Fang, Qiming Ouyang, Zhi Zheng, Jin Wang

**Affiliations:** Department of Oral Maxillofacial Surgery, Fujian Medical University Union Hospital, Fuzhou, China

**Keywords:** hemiglossectomy, superficial circumflex iliac artery perforator flap, tongue reconstruction, oral maxillofacial reconstruction, tongue cancer

## Abstract

**Objective:** Therapeutic evaluation of applying superficial circumflex iliac artery perforator (SCIP) flap in tongue reconstruction after hemiglossectomy.

**Methods:** We retrospectively collected a total number of 14 patients who received SCIP flap reconstruction for tongue defects at the Fujian Medical University affiliated Union Hospital, between November 2016 and November 2020. We evaluated the clinical features of the flap, analyzed the postoperative functions and esthetic effects after 6 months of the tongue reconstruction.

**Results:** All the SCIP flaps survived successfully after the operation, the recipient site tongue bodies were well reconstructed 6 months after the surgery. Patient speech and swallowing functions were well recovered during the follow-up. The scars in the donor site were concealed with limited numbness.

**Conclusions:** With the advantages of the hidden donor site and aesthetic benefits, the SCIP flap is a reliable option for the reconstruction of the tongue defect after hemiglossectomy.

## Introduction

Tongue cancer is the most common type of oral malignant tumor, which makes up 40–50% of all oral cavity cancers (1). Surgical resection to remove the lesion is the conventional strategy to treat such neoplasms (2). However, surgical treatment usually results in soft tissue defects, and esthetic and functional deficiencies, leading to a decline in the quality of life. With the advancement in microvascular tongue reconstruction, surgeons can restore the oral function of patients by utilizing different flaps (3).

According to the classification of tongue resection for tumor removal, we can define tongue defects as subtotal, total glossectomy, and hemiglossectomy (3). For subtotal glossectomy defects, anterolateral thigh perforator flaps (ALTF) or anterolateral thigh myocutaneous flaps are recommended (4); for total glossectomy defects, pentagonal anterolateral thigh myocutaneous flaps are preferred (5); for hemiglossectomy defects, radial forearm free flaps (RFFF), ALTF, and superficial circumflex iliac artery perforator (SCIP) flaps are the available options (6). In this study, we focused on the testimony for hemiglossectomy reconstruction.

In the restoration of partial tongue defects, RFFF is selected due to its pliability and long pedicle; however, the morbidity of its donor site remains a major concern (7). As a substitution, ALTF has received more attention in recent years due to its adaptability and reduced donor site morbidity, although it is only suitable for patients with thin skin (8). An alternative feasible choice of the flap is SCIP, with many benefits, such as superficial and shorter dissection for pedicle vessel, reduced flap operative time, minimal donor site morbidity, customizable thickness for tissue augmentation, and a concealed donor site with an inconspicuous scar that can be hidden by underwear (9). In this study, based on the postoperative observation and evaluation, we report SCIP as the most elegant choice for hemiglossectomy defect reconstruction.

## Materials and Methods

### Patients

This clinical study was conducted at the Oral & Maxillofacial Department in the Fujian Medical University affiliated Union Hospital from November 2016 to November 2020. Ethics permission was approved by the Institutional Clinical Research Supervision Committee. Informed consent agreements for tongue reconstruction were collected from each patient. A total of 14 patients (six men and eight women) were recruited and scheduled for SCIP flap transfer after hemiglossectomy and neck dissection. The patient information is summarized in Table 1.

**Table 1:** Patient characteristics.

**Case**	**Gender**	**Age**	**BMI**	**Waist(cm)**	Primary tumor^*^	**Tumor stage (TNM)**	**Follow-up (Months)**	**Recurrence**
1	Female	25	21.5	73	SCC	T2N1M0	18	Absence
2	Female	32	21.7	74.2	SCC	T2N0M0	12	Absence
3	Male	38	24.6	81.5	SCC	T2N1M0	12	Absence
4	Male	41	23.8	79.2	SCC	T2N1M0	15	Absence
5	Female	43	26.3	86.2	SCC	T2N1M0	9	Absence
6	Male	47	24.2	82.4	SCC	T2N1M0	6	Absence
7	Female	48	23.4	78.6	SCC	T2N0M0	18	Absence
8	Female	55	26.2	85.9	SCC	T2N2M0	9	Absence
9	Male	56	27.1	88.3	SCC	T2N0M0	9	Absence
10	Female	60	29.1	92.8	SCC	T2N1M0	6	Absence
11	Female	37	22.5	76.6	SCC	T2N0M0	12	Absence
12	Male	65	21.8	74	SCC	T2N1M0	12	Absence
13	Male	51	23.9	80.2	SCC	T2N1M0	9	Absence
14	Female	42	25.7	84.2	SCC	T2N0M0	6	Absence

* *SCC, Squamous cell carcinoma*.

The average age of the 14 patients was 45.7 years (range 25–65 years), and with an average body mass index of 24.4. In this patient cohort, we have four overweight women patients and one overweight man patient. Histological diagnosis of all 14 patients was squamous cell carcinoma, five of whom had a tumor staged as T2N0M0, eight of whom were at stage T2N1M0, and only one of whom was at tumor stage T2N2M0. The standard of tumor stage we referred to was defined by the 2016 Union for International Cancer Control TNM classification of lip and oral cavity malignant tumors (10). No tumor recurrence was diagnosed within the follow-up periods with a range of 6–18 months.

### Flap Preparation

Patients underwent hemiglossectomy and ipsilateral cervical lymph node dissection before the reconstruction. Doppler ultrasound examinations were performed for pinpointing the location of cutaneous perforators of the superficial circumflex iliac artery (SCIA) before the surgery. In the meantime, the projection lines of the branches of the SCIA on the body surface were drawn according to the blood signal, with patients lying in the supine position. According to the evaluation of the defect dimension and surrounding tissues, each flap was designed centered on the perforator of the SCIA marked preoperatively. The femoral artery was labeled by identifying the midpoint of the line between the anterosuperior iliac spine and the symphysis pubis, also acknowledged as the center of the inguinal skin crease, where the femoral artery can be readily palpated (11).

The first incision was made on the medial edge of the flap, extending the incision along the inguinal crease ~4–6 cm. The incision was carried down the skin and subcutaneous tissue, and the superficial circumflex iliac vein (SCIV) could be identified in the subcutaneous fat layer, as the SCIV typically runs parallel to the inguinal ligament. Careful dissection to open the superficial fascia and to avoid accidental bleeding was performed. The superficial branch of the SCIA could be identified in the fatty tissue in the deep fascia and could be traced back to the main trunk of the SCIA. In case the superficial branch was not obvious, the depth of the incision was made through the fat layer to expose the deep fascia to locate the deep branch of the SCIA. Once the SCIA was determined, meticulous dissection was performed proximally and distally until an appropriate length was achieved. During the operation, careful dissection of the blood vessel is necessary to make sure the vascular pedicle is not too bloated, this way we can lengthen the vascular pedicle.

### Flap Indicators

Among these 14 patients, the flap size ranged from 45 to 67.5 cm^2^, the average flap thickness was 1.07 cm. The mean length of the arterial pedicle was 8.01 cm. The mean length of the venous pedicle was 8.30 cm. The mean diameter of the artery was 0.78 mm. The mean caliber of the vein was 1.04 mm. Details of the flap indicators are listed in Table 2.

**Table 2:** Detailed information of characteristics of SCIP flaps.

**Case**	**Flap size (cm)**	**Flap thickness (cm)**	**Arterial pedicle length (cm)**	**Venous pedicle length (cm)**	**Caliber of artery (mm)**	**Caliber of vein (mm)**
1	6 * 8	0.7	8	8	0.6	0.9
2	6 * 8	0.8	7.5	8	0.7	1
3	7 * 8	1.1	8.5	8.5	0.9	1.4
4	6 * 7.5	0.9	7.2	7.5	0.8	0.8
5	7.5 * 9	1.3	9	9	1.1	1.3
6	7 * 8	1	9.2	9.2	0.6	0.9
7	6.5 * 7	0.9	7	7.2	0.8	1.1
8	6.5 * 7	1.3	8.3	8.5	0.7	1.2
9	6 * 7.5	1.4	8.4	8.6	0.7	0.9
10	6.5 * 9	1.5	8.6	9.1	0.8	1.2
11	7 * 7	1.0	7.5	7.8	0.8	1.0
12	6.5 * 8	0.8	8.2	8.8	0.9	1.1
13	6.5 * 7.5	1.1	7.1	7.6	0.8	0.9
14	7 * 8	1.2	7.7	8.2	0.7	0.9

### Defect Reconstruction

The SCIP flap was transferred to the defect recipient site, making sure to place its long axis parallel to the long axis of the tongue. Microvascular end-to-end anastomosis was performed between the SCIA and the distal segment of the superior thyroid artery. An appropriate recipient vein was carefully selected among the superior thyroid vein, the middle thyroid vein, and the facial vein to find a vessel with comparable caliber to anastomose with the superficial cutaneous vein in a homolateral manner to the defect side. The donor site was directly closed and a negative pressure suction drain was placed at the end of the surgery.

### Postoperative Management

After surgery, patients were made to rest in bed for a couple of days and to avoid over oscillating their heads. Patients were routinely treated with papaverine injection to prevent vasospasm, and low-molecular-weight heparin (LMWH) sodium was injected subcutaneously to prevent blood clotting.

### Evaluation of Donor Site, Recipient Site Recovery

Postoperative assessments of flap survival included examination of the pigment, dermatoglyphics of the flap, and evaluation of the capillary refilling degree. Measurement of speech and swallowing functions were also performed after a regular 6-month follow-up period. Specifically, a mandarin scoring system was applied to a speech-language pathological test (12). The pronunciation intelligibility degree = number of correct pronunciations/total number of characters in vocabulary table * 100%. The resulting scores can be interpreted as follows: normal (97%), slightly impaired (71–96%), moderately impaired (36–70%), and largely impaired (0–35%). Furthermore, a swallowing function assessment was also measured based on the types of patient diet with the subsequent categories: regular diet, soft diet, semiliquid diet, and liquid diet.

### Results

Among the cohort of 14 patients, all flaps survived successfully without partial necrosis. After 6 months of regular follow-up, all patients had intelligible speech with either normal or slightly impaired test results. Eleven patients could manage a soft diet, while the other three patients could manage a regular diet. All of our patients were satisfied with the concealed donor site and good restoration of the functional and aesthetic properties of the recipient site. Regarding the wound healing stages in these 14 patients, both the recipient and donor sites were in stage I without complications. The detailed follow-up information is listed in Table 3.

**Table 3:** Functional evaluation of SCIP flap reconstruction after 6-month follow-up.

**Case**	**Flap survival**	**Healing of donor site**	**Speech**	**Swallowing**
1	Good	Stage I	Normal	Regular
2	Good	Stage I	Normal	Regular
3	Good	Stage I	Normal	Soft
4	Good	Stage I	Slightly impaired	Soft
5	Good	Stage I	Normal	Regular
6	Good	Stage I	Normal	Soft
7	Good	Stage I	Slightly impaired	Soft
8	Good	Stage I	Slightly impaired	Soft
9	Good	Stage I	Slightly impaired	Soft
10	Good	Stage I	Slightly impaired	Soft
11	Good	Stage I	Normal	Soft
12	Good	Stage I	Normal	Soft
13	Good	Stage I	Slightly impaired	Soft
14	Good	Stage I	Slightly impaired	Soft

### Case 1

**Figure 1:**
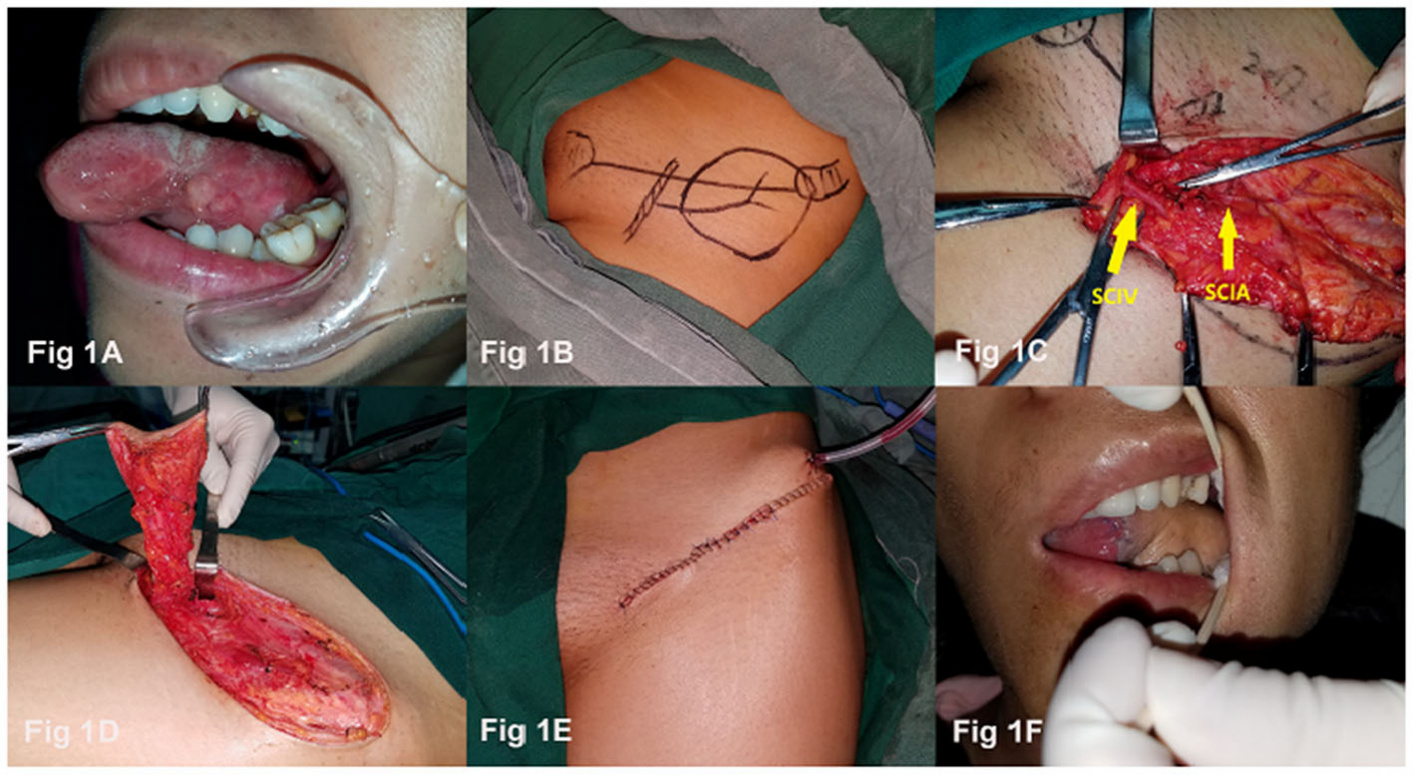
SCIP flap preparation and reconstruction process.

A 32-year-old woman presented with T2N0M0 squamous cell carcinoma of the left tongue (Figure 1A). The patient underwent hemiglossectomy and ipsilateral lymph node dissection; the SCIP flap was designed simultaneously with the operation on her oral region (Figure 1B). In the surgical procedure, after identifying the SCIV, the SCIA was also exposed (Figure 1C), then a 6 × 8 cm SCIP flap was harvested with a 7.5 cm arterial pedicle and an 8 cm venous pedicle (Figure 1D). The SCIA and superficial cutaneous vein were anastomosed to the corresponding superior thyroid artery and facial vein homolateral to the defect side. The donor site was carefully closed and a negative pressure suction drain was placed (Figure 1E). A postoperative oral image was taken at a 1-week examination (Figure 1F). After 6 months of regular follow-up, the contour and the function of the reconstructed tongue were satisfactory and the donor site scar was concealed with ordinary clothing.

## Discussion

One of the most challenging surgeries after the removal of lesions in oral cancers is tongue reconstruction (13). As the critical sensory organ for speech and swallowing, the tongue plays a central role in the postsurgical day-to-day life of patients while eating and talking. Surgical restoration of tongue defects is directly correlated to the overall quality of life of patients (14). Previously, the most common flap for tongue reconstruction was RFFF, as it offers a steady pattern of vascular anatomy and a pliable skin paddle (15). However, there are downsides of the RFFF, which are also quite obvious from the aesthetic perspective (16). Previous research has been carried out to identify an alternative donor site for replacing the deficient area after glossectomy (17). Inspired by the first free groin flap based on SCIA for limb reconstruction reported by Daniel et al. (18), many overtures have been made to explore the application of a groin flap. However, it took two decades for scientists to resolve the weakness of harvesting a groin flap due to its high variations on the trunk of the SCIA and its superficial branch (19). Resounding evidence of the benefits of utilizing SCIP in extremities reconstruction was first delineated by Koshima (9), which paved the way for the repopularization of using SCIP in plastic surgery. The pragmatic advantages of SCIP in defect reconstruction include superficial dissection for the pedicle vessel with reduced flap harvest time, customizable thickness, the obtainability of a large cutaneous vein for an intravenous drainage system, and minimum invasiveness at the donor site (20). From there forward, SCIP has been widely applied to reconstructions of limbs, penis, ears, and other head and neck defect (21).

In this practice, we believe that SCIP is a promising flap with many irresistible merits in tongue defect reconstruction after the patient goes through hemiglossectomy. First of all, from an aesthetic perspective, it is more favorable among younger patients. Ma et al. (22) reported almost all of their nine cases of patients (average age of 44.7 years) picked the SCIP flap as their first option when different flap choices were introduced, and the same decision-making process occurred among our patients, with an average age of 45.7 years. Second, as a hair-less skin paddle, SCIP can reduce foreign body sensation during the rehabilitation of patients. A significant but rare side effect of radial forearm flaps or anterolateral thigh flaps is potential hair growth at the recipient site, which might be due to the occurrence of hair follicles in the donor sites (23, 24). Although a preventive treatment with laser to epilate hairs from the donor site can be applied, the risk of an intraoral hairy flap might result in a very grotesque complication which could bring persistent difficulties to the postoperative life of patients. In this light, SCIP is a considerably promising option by preventing this incongruous complication from happening in the very beginning. Third, with a mean thickness of 1.07 cm, the SCIP in our 14 cases provided complementary volume and texture to the recipient site, which assures the recovery of the contour of the defect and satisfactory swallowing and speech functions. In addition, advanced improvement of sensate SCIP based on lateral cutaneous branches of the intercostal nerve has been developed and reported in several cases (25). Although reports on sensate SCIP and its clinical applications are still limited, this creates a future direction for SCIP refinement, which could provide great potential in improving the quality of life of patients after the reconstruction of tongue defects.

The major concern about SCIP is the variability of the perforator vasculature. However, this issue can be properly handled by preoperative Doppler ultrasound examinations. In our practice, we found the ubiquitous existence of only one dominant perforator, the superficial branches and deep branches of the SCIA have a complementary relationship (20). Patients with a strong and long deep branch of the SCIA generally have a short superficial branch, and a deep branch is considered as the dominant perforator; when patients have short and small deep branches of the SCIA, usually a long and large superficial branch can be identified, in this case, the superficial branch is the dominant perforator. We suggest including both deep and superficial branches of the SCIA to guarantee a sufficient blood supply for the flap.

In addition, SCIP flaps have a diameter of the artery that is smaller compared to other conventional flaps. This results in difficulties in searching for appropriate recipient vessels in the neck for a safe end-to-end anastomosis. Thus, a careful selection of the flap choice should be thoroughly discussed. During the dissection, we also tried to preserve the length of the recipient blood vessel to extend the vascular pedicle so that the vascular pedicle will not be strained. If the desired recipient artery with an adequate caliber is not found, the anastomosis could be performed in end-to-side fashion or supermicrosurgery skills might be necessary.

In conclusion, our study suggests that a SCIP flap is a reliable flap and can be a good alternative for the restoration of a defect following hemiglossectomy. With many functional and aesthetic advantages, SCIP flaps for tongue defect reconstruction are promising and will continue to benefit patients in the future.

## Data Availability Statement

The original contributions presented in the study are included in the article/supplementary material, further inquiries can be directed to the corresponding author/s.

## Ethics Statement

The studies involving human participants were reviewed and approved by the Institutional Clinical Research Supervision Committee of Oral & Maxillofacial department in the Fujian Medical University affiliated Union Hospital. The Ethics Committee reference number is: 2020ky0120. The patients/participants provided their written informed consent to participate in this study. Written informed consent was obtained from the individual(s) for the publication of any potentially identifiable images or data included in this article.

## Author Contributions

YF, QO, ZZ, and JW conceptualized the study, performed the surgeries, carried out the analyses, and wrote the manuscript. YF contributed to carrying out the analyses. All authors contributed to the article and approved the submitted version.

## Conflict of Interest

The authors declare that the research was conducted in the absence of any commercial or financial relationships that could be construed as a potential conflict of interest.

## Publisher's Note

All claims expressed in this article are solely those of the authors and do not necessarily represent those of their affiliated organizations, or those of the publisher, the editors and the reviewers. Any product that may be evaluated in this article, or claim that may be made by its manufacturer, is not guaranteed or endorsed by the publisher.
